# AVE 0991 Attenuates Pyroptosis and Liver Damage after Heatstroke by Inhibiting the ROS-NLRP3 Inflammatory Signalling Pathway

**DOI:** 10.1155/2019/1806234

**Published:** 2019-08-19

**Authors:** Ming Zhang, Xintao Zhu, Huasheng Tong, Anni Lou, Yue Li, Yang Li, Lei Su, Xu Li

**Affiliations:** ^1^Department of Emergency Medicine, Nanfang Hospital, Southern Medical University, Guangzhou, Guangdong, China; ^2^Department of Intensive Care Unit, The Sixth Affiliated Hospital of Guangzhou Medical University, Qingyuan People's Hospital, Qingyuan, Guangdong, China; ^3^Department of Gastroenterology, Nanfang Hospital, Southern Medical University, Guangzhou, Guangdong, China; ^4^Department of Intensive Care Unit, General Hospital of Southern Theatre Command, Southern Medical University, Guangzhou, Guangdong, China

## Abstract

We previously demonstrated that angiotensin-(1-7) (Ang-(1-7)), an essential endocrine factor, inhibits the NLRP3 inflammasome by regulating reactive oxygen species (ROS) in fibrotic livers. We also demonstrated that the NLRP3 inflammasome contributes to the liver damage induced by pyroptosis after heatstroke. However, the role of Ang-(1-7) in the hepatocytes under heat stress remains uncertain. We aimed to examine the change in angiotensin peptides in the livers affected by heatstroke and the effect on the ROS-NLRP3 inflammatory signalling pathway.* In vivo*, increased angiotensin II (Ang II) and decreased Ang-(1-7) in the serum of heatstroke patients suffering from hepatic dysfunction were observed. The change in angiotensin peptides was considered a potential biomarker that could be used to predict hepatic dysfunction. Enhanced Ang II and attenuated Ang-(1-7) levels were also observed in the liver tissue of heatstroke rats, which were consistent with their receptors and converting enzymes. Hepatic damage associated with increased ROS and protein expression levels of NOX4, NLRP3, caspase-1, and IL-1*β* was attenuated by AVE 0991, an analogue of Ang-(1-7).* In vitro*, pyroptosis, characterized by activated caspase-1 and IL-1*β*, was observed in hepatocytes under heat stress, which was enhanced by Ang II and attenuated by antioxidants, NOX4 siRNA, and AVE 0991. In summary, AVE 0991 attenuates pyroptosis and liver damage induced by heat stress by inhibiting the ROS-NLRP3 inflammatory signalling pathway.

## 1. Introduction

Liver injury, which is a serious complication of heatstroke, may develop into refractory liver failure and lead to death [1]. To date, the mechanism by which heatstroke causes liver injury remains uncertain. Despite temporary and invasive replacement therapy, there is still no definite therapy to treat liver injury [2]. Pyroptosis is characterized by the activation of caspase-1, resulting in programmed cell death and extensive release of inflammatory cytokines, including IL-1*β* [3, 4]. NOD-like receptor family pyrin domain containing 3 (NLRP3), which is an intracellular pattern recognition receptor, may contribute to pyroptosis by assembling into an inflammasome [5]. Our previous study suggested that NLRP3-dependent pyroptosis is responsible for liver injury after heatstroke, which is one of the few known mechanisms [6]. However, the precise signalling pathway and favourable negative regulation of this process remain elusive.

ROS, which are crucial stimuli of the NLRP3 inflammasome, serve as a potential target for the negative regulation of pyroptosis [7]. Evidence has indicated that excessive amounts of ROS are produced during heat stress, but the precise source of ROS production is uncertain [8]. However, over-inhibition of ROS that serve as signalling molecules may obstruct their favourable role, which has been demonstrated by the uncertain efficacy of antioxidants in inflammatory diseases [9]. Therefore, there is a need for controllable inhibition of the uncertain source that produces excessive amounts of ROS as a result of heatstroke.

The renin-angiotensin system (RAS), which is widely distributed in addition to the cardiovascular system, plays a crucial role by regulating ROS production. The change in angiotensin peptides, which is the main components of RAS, has been observed in various diseases accompanied by unfavourable ROS derivatives [10]. We previously demonstrated that neutralizing this change using an endogenous antagonist rescued the over-production of ROS, resulting in the decrease in NLRP3-dependent damage in cirrhotic rats (Cai et al., 2015). Furthermore, our previous study initially revealed evidence regarding the change in angiotensin peptides in heatstroke rats [11]. However, the exact condition and role of angiotensin peptides in livers affected by heatstroke remain unclear. Therefore, we hypothesized that changes contributed to the over-production of ROS and NLRP3-dependent liver injury as a result of heatstroke.

In this study, we aimed to investigate the change in angiotensin and the role of angiotensin in livers subjected to heatstroke. We demonstrated that AVE 0991 attenuates pyroptosis and liver damage after heatstroke by inhibiting the ROS-NLRP3 inflammatory signalling pathway.

## 2. Materials and Methods 

### 2.1. Reagents

Angiotensin II (Ang II), diphenyleneiodonium (DPI) and catalase (CAT) were purchased from Sigma-Aldrich (St. Louis, MO, USA). AVE 0991 was purchased from Apexbio (Houston, TX, USA). Losartan was purchased from Selleck Chemicals (Houston, TX, USA). Small interfering RNA (siRNA) targeting NOX4 was purchased from GenePharma (Shanghai, China).

### 2.2. Patients

Our clinical prospective study was conducted at the General Hospital of Southern Theatre Command between April 2013 and May 2018. The study conformed to the ethical guidelines and was approved by the local ethics committee. Written informed consent forms were signed by the study participants. Heatstroke was diagnosed by an elevated core temperature above 40°C and abnormalities of the central nervous system due to a hot and humid environment [12]. Intense physical exercise before onset was a contribution to all the participants. Their hepatic dysfunction was dynamically monitored within one week after onset. Total bilirubin exceeding 34 *μ*mol/L was defined as the criterion of acute hepatic dysfunction [13, 14]. The blood samples from heatstroke patients obtained on the day of admission were retrospectively analyzed. Serum was stored at −80°C until it was analysed, and repeated freeze-thaw cycles were avoided. Exclusion criteria include comorbidities (hypertension, infection and chronic organ dysfunction) and unavailable blood samples on the first day of onset. This observation hardly interfered with the standard treatment procedures.

### 2.3. Animals

Adult male Sprague-Dawley rats, weighing approximately 200-220 g, were provided by the Laboratory Animal Center of Southern Medical University (Guangzhou, China). The rats were provided standard chow and water and housed at 22-24°C for one week before the experiment. They were habituated to a climate chamber for one week before the experiment. The protocols for the animal experiments were approved by the Animal Care and Use Committee of General Hospital of Southern Theatre Command.

### 2.4. A Heatstroke Rat Model of Liver Damage

The autonomous rats were placed into the climate chamber without food and water, where the temperature was then increased to 39.5±0.2°C and the relative humidity was 60±5%. The systolic blood pressure of the tail artery and the core (rectal) temperature were monitored as described in our previous study [6]. The time-point at which the SBP decreased from the peak level and the core temperature exceeded 42°C was used as a reference point of heatstroke onset [15]. The heatstroke rats were removed from the chamber, cooled by ice, and provided with water immediately. When their core temperature decreased to the baseline and consciousness was recovered, the rats were returned to the 22-24°C environment with free access to water. The sham rats participated in the same process without heat stress. AVE 0991 (600 *μ*g/kg) was injected i.p. into the rats in the AVE group, and the liver tissue and blood samples were harvested after 9 h (n=9 per group).

### 2.5. Measurement of H_2_O_2_, GSH, ALT, and LDH

Hydrogen peroxide (H2O2) content (Nanjing Jiancheng Bioengineering Institute, China), GSH content (Beyotime Biotechnology, China), ALT in serum (Nanjing Jiancheng Bioengineering Institute, China) and LDH in hepatocyte supernatant (Nanjing Jiancheng Bioengineering Institute, China) were analysed using commercial kits according to the manufacturer's instructions.

### 2.6. ELISA

The concentrations of Ang II and Ang-(1-7) were analysed using enzyme-linked immunosorbent assay (ELISA) kits (Cusabio Biotech, Wuhan, China). ACE (angiotensin converting enzyme) and ACE2 were analysed using ELISA kits purchased from USCN company (Wuhan, China). Serum samples were obtained from whole blood allowed to clot and centrifuged at low temperature. The liver tissue of rats were minced into small pieces and then homogenized for protein extraction in the fresh lysis buffer (Cusabio Biotech). The samples were evaluated after being sonicated and centrifuged strictly as suggested by instructions. The repeated freeze/thaw cycles were avoided.

### 2.7. Real-Time qPCR

Total RNA in the liver tissue was extracted with Trizol reagent (TIANGEN, Beijing, China) and reverse transcribed with PrimeScript™ RT Master Mix (Takara, Tokyo, Japan). Real-time qPCR was performed using SYBR Premix Ex Taqt™ II (Takara, Tokyo, Japan). The following primers were used: GAPDH Primer (forward: 5′-AGTTCAACGGCACAGTCAAG-3′; reverse: 5′-TACTCAGCACCAGCATCACC-3′); AT1 receptor primer (forward: 5′-TGTCATGATCCCTACCCTCTAC-3′; reverse: 5′-GCCACAGTCTTCAGCTTCAT-3′); and MAS receptor primer (forward: 5′-CGGTCTATATCACCCACTTGTC-3′; reverse: 5′-GGCCAGAAGAGAGTTCATAGTC-3′);

### 2.8. Histological and Immunohistochemistry Analysis

Haematoxylin and eosin (HE) staining, marrow peroxidase (MPO) staining and immunohistochemical (IHC) staining were conducted on paraffin-embedded liver tissues and observed by microscopy. HE-stained sections were scored by a pathologist according to a previously described method [16]. IHC staining of the AT1 receptor, the MAS receptor, NOX4, NLRP3, IL-1*β* p17, and caspase-1 p10 (1:100 dilution; Abcam (Cambridge, MA, USA)) was performed.

### 2.9. Cell Culture

The HBL3A cell line was provided by the cell bank of the Chinese Academy of Sciences (Shanghai, China). The cells were cultured in plates with medium comprising 20% foetal bovine serum (Gibco, CA, USA). The cells were stimulated by heat stress for 1 h at 43°C and 5% CO_2_ and were moved back to the incubator at 37°C and 5% CO_2_, with or without agents (Ang II, AVE 0991, DPI (10^-5 ^M), or CAT (10 mM)) added to the supernatant for an additional 9 h.

### 2.10. Small Interfering RNA (siRNA) Transfection

HBL3A cells were transfected with siRNA sequences targeting the protein NOX4 (sense: 5′-GGGCCAGAAUACUACUACATT-3′; antisense: 5′-UGUAGUAGUAUUCUGGCCCTT-3′), and the efficiency of transfection was 70%. The transfection protocol was performed according to the manufacturer's instructions.

### 2.11. Analysis of Intracellular ROS by Flow Cytometry

A DCFH-DA reactive oxygen detection kit was purchased from Beyotime Biotechnology (Shanghai, China). The DCFH-DA fluorescent probe oxidized by reactive oxygen species was analysed using a flow cytometer (Guava 5HT, Millipore, MA, USA).

### 2.12. Pyroptosis Analysis by Flow Cytometry

To examine pyroptosis of HBL3A cells, we analysed activated caspase-1 using a FLICAR 660 detection kit (FLICA 660-YYAD-FMK, ImmunoChemistry Technologies, Bloomington, MN). The samples were analysed using flow cytometry (BD LSRFortessaTM X-20. BD Biosciences, San Jose, CA) and FlowJo analytical software (Tree Star, Ashland, OR, USA).

### 2.13. Immunofluorescent Cytochemistry

After fixation with paraformaldehyde, the cells were incubated with antibodies against NLPR3 and caspase-1 (1:200; Abcam). The secondary antibodies included Cy3-conjugated anti-goat or fluorescein isothiocyanate-conjugated anti-rabbit antibodies.

### 2.14. Western Blotting

Protein expression in liver tissue or HBL3A cells was analysed by western blotting. The primary antibodies included *β*-actin (Cell Signaling Technologies, 1:1000), MAS receptor (Thermo, 1:2000), AT1 receptor, NOX4, NLRP3, IL-1*β* p17, andcaspase-1 p10 (Abcam, 1:1000). All western blots were repeated at least three times.

### 2.15. Statistics

Continuous variables are presented as the mean ± standard deviation or the median (the 25th and 75th percentile) based on whether the variables met the Kolmogorov-Smirnov criteria. Categorical variables are presented as frequencies. Student's t-test, Mann-Whitney U test, and *χ*^2^ test were used to investigate differences between the groups. A receiver operating characteristic (ROC) curve was used to assess the accuracy of the markers. The calibration was evaluated with the Hosmer-Lemeshow *χ*^2^ statistic. The comparison of survival curves was analysed with log-rank test. Statistical significance was defined as a* P*-value <0.05. All analyses were performed using SPSS 19.0 for Windows (SPSS Inc., Chicago, IL, USA).

## 3. Results

### 3.1. Increased Ang II and Decreased Ang-(1-7) in the Serum of Heatstroke Patients with Liver Dysfunction

**Figure 1:**
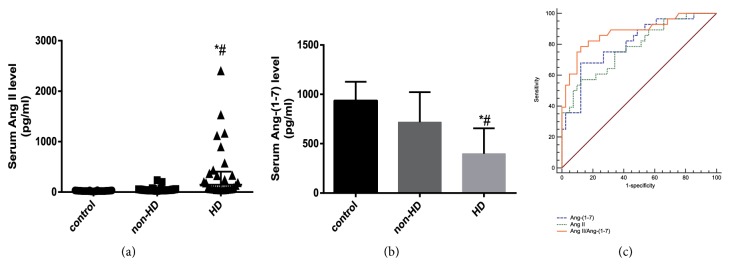
*Elevated angiotensin II and descended angiotensin-(1-7) in heatstroke patients with liver dysfunction*. Angiotensin II (Ang II) content (a) and angiotensin-(1-7) (Ang-(1-7)) content (b) in the serum of healthy controls (control), heatstroke patients without liver dysfunction (non-HD) and heatstroke patients with liver dysfunction (HD). The assays were performed in triplicate. *∗P*<0.05 compared with the control group and #*P* <0.05 compared with the non-HD group. (c) The receiver operating characteristic curves of Ang II, Ang-(1-7) and Ang II/Ang-(1-7) regarding the occurrence of liver dysfunction within one week after heatstroke. The AUC of three curves were significantly larger than 0.5 (*P*<0.001).

Sixty-nine patients were enrolled in this study. The patients were all young male adults with a median age of 21.0 (19.0, 27.0) years. A total of 53 healthy men younger than 30 years of age were also enrolled. According to whether hepatic dysfunction occurred within one week after admission, 28 patients (40.6%) were divided into the hepatic dysfunction (HD) group, and the other 41 patients (59.4%) were divided into the non-hepatic dysfunction (non-HD) group. The patients in the HD group were associated with increased organ support therapy (invasive mechanical ventilation, haemopurification, haemodynamic support), prolonged length of ICU stay and increased mortality (Table 1). An increased level of Ang II and a decreased level of Ang-(1-7) were observed in the HD group compared to the healthy control and non-HD groups (Table 1 and Figures 1(a) and 1(b)).

### 3.2. Ang II, Ang-(1-7), and Ang II/Ang-(1-7) Were Potential Biomarkers for Hepatic Dysfunction in Heatstroke Patients

The ROC analysis showed that the Ang II and Ang-(1-7) levels upon admission predicted the occurrence of liver dysfunction within one week. The ratio of Ang II/Ang-(1-7) had excellent discrimination with a larger area under the curve (Table 2 and Figure 1(c)). Ang II, Ang-(1-7), and Ang II/Ang-(1-7) all passed the subsequent Hosmer-Lemeshow *χ*^2^ test, suggesting acceptable calibration (Table 3).

### 3.3. Physiological Response to Heatstroke

Heatstroke induce typical physiological responses in rats such as blood pressure, core temperature, weight and so on (Table 4). Despite rapid cooling and water feeding to rescue death from circulatory failure, there were still persistent nervous system dysfunction and hypotension within hours after heatstroke.

### 3.4. Enhanced Function of Ang II and Attenuated Function of Ang-(1-7) in Heatstroke Rats

**Figure 2:**
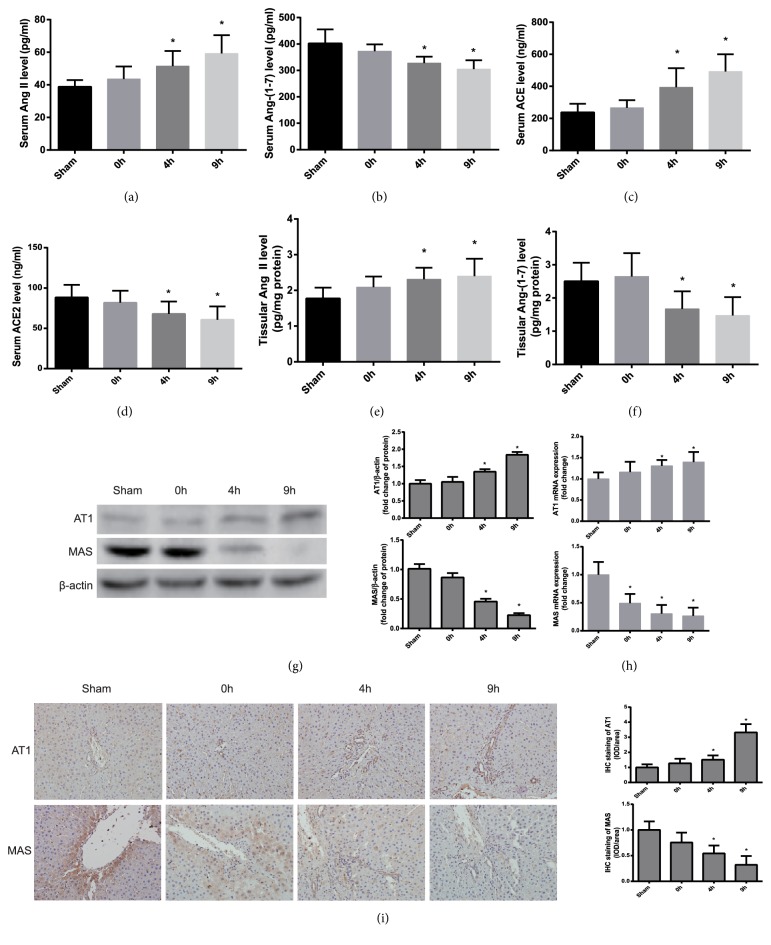
*The expression of renin-angiotensin system after the onset of heatstroke in rats*. (a–i) Samples of serum and liver sections were obtained from the sham rats and heatstroke rats at the time-point of heatstroke onset (0 h) and 4 and 9 h after heatstroke onset. The serum levels of Ang II (a), Ang-(1-7) (b), ACE (c), and ACE2 (d) were analysed by ELISA. The liver contents of Ang II (e) and Ang-(1-7) (f) were analysed by ELISA. (g) Protein expression of the AT1 receptor and the MAS receptor in liver tissue was analysed by western blotting. The quantification of the relative protein expression is shown in the graph (right). (h) Real-time PCR analysis of the AT1 receptor and the MAS receptor mRNA expression in liver tissue. (i) Immunohistochemical staining was performed to analyse AT1 and MAS protein expression. *∗P*<0.05 compared with the control group. The data are presented as the mean ± SD. All of the assays were performed in triplicate. ACE: angiotensin converting enzyme; ACE2: angiotensin-converting enzyme 2; AT1: angiotensin II type 1 receptor.

The effectors and receptors of two main axes in RAS, the ACE-Ang II-AT1 receptor and the ACE2-Ang-(1-7)-MAS receptor were analysed in heatstroke rats. The results indicated that greater expression of Ang II and lower expression of Ang-(1-7) were demonstrated in the serum and liver tissue of heatstroke rats compared with those of controls, which was consistent with their receptors (AT1 and MAS) in the tissue and converting enzymes (ACE and ACE2) in the serum (Figures 2(a)–2(i)), suggesting that the function of Ang II was enhanced and that of Ang-(1-7) was attenuated under heat stress.

### 3.5. AVE 0991 Protected against Liver Injury Induced by Ang II and Heat Stress* In Vivo* and* In Vitro*

**Figure 3:**
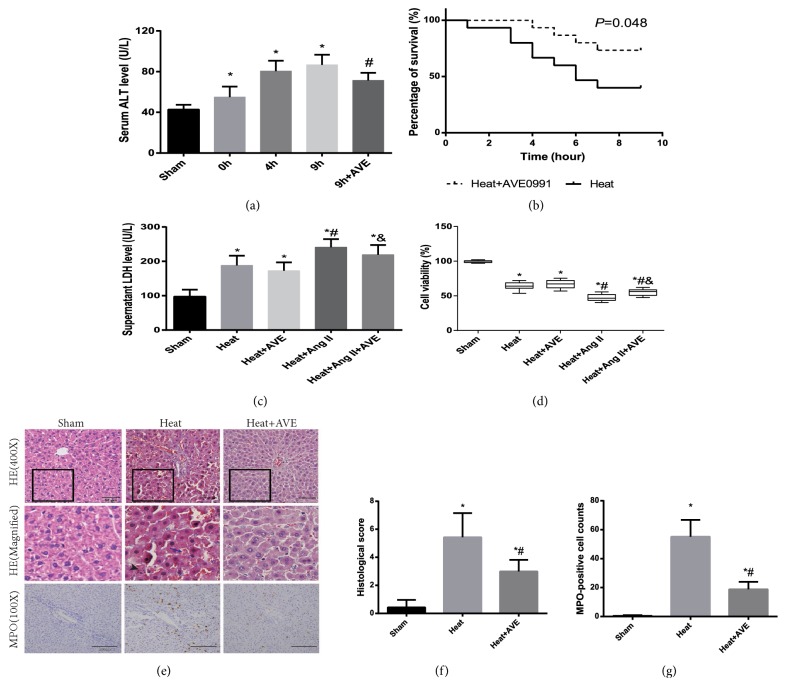
*The effect of AVE 0991 on the hepatic damage induced by heatstroke and Ang II*. (a) Hepatic damage evaluated by analysing serum ALT at the time-point of heatstroke onset (0 h) and 4 and 9 h after heatstroke onset. The rats in the 9 h+AVE group were injected with AVE 0991 at the time-point of heatstroke onset and sacrificed after 9 h. n = 9 per group. *∗P*<0.05 compared with the sham group. #*P*<0.05 compared with the 9 h group. (b) The survival curves of rats with or without AVE 0991 injection after heatstroke (*P*=0.48). n=12 per group. (c) Hepatocellular damage was evaluated by analysing LDH in the supernatant. (d) Cell viability was evaluated using a CCK-8 assay. The sham group was not subjected to heat stress. Ang II and/or AVE 0991 were added to the BRL-3A supernatant for an additional 9 h after heat stress.*∗P*<0.05 compared with the sham group. #*P*<0.05 compared with the heat group. &*P*<0.05 compared with the heat+Ang II group. (e) (top) HE staining of liver sections from rats. Heat group: 9 h after heatstroke onset. Black rectangles indicate the area of magnification. Scale bar: 50 *μ*m. Magnification of HE-stained liver section images (middle). The black arrow indicates necrosis. The blue arrow with a long handle indicates haemorrhage. MPO staining of liver sections (bottom). Scale bar: 200 *μ*m. (f) Histological scores of liver sections. (g) MPO-positive cell counts of liver sections. n=6 per group. *∗P*<0.05 compared with the sham group. #*P*<0.05 compared with the heat group. The data are presented as the mean ± SD. All of the assays were performed in triplicate. HE: Haematoxylin-eosin; MPO: myeloperoxidase.

Serum ALT content, HE staining and MPO staining showed that liver injury induced by heatstroke was attenuated by AVE 0991 in rats. AVE 0991 improved the survival rate of rats after heatstroke compared with placebo.* In vitro*, the LDH level of the supernatant and CCK-8 assay results suggested that heat stress caused hepatocellular damage, which was enhanced by Ang II and rescued by AVE 0991 (Figure 3).

### 3.6. AVE 0991 Attenuated Ang II-Induced ROS Mediated by NOX4

**Figure 4:**
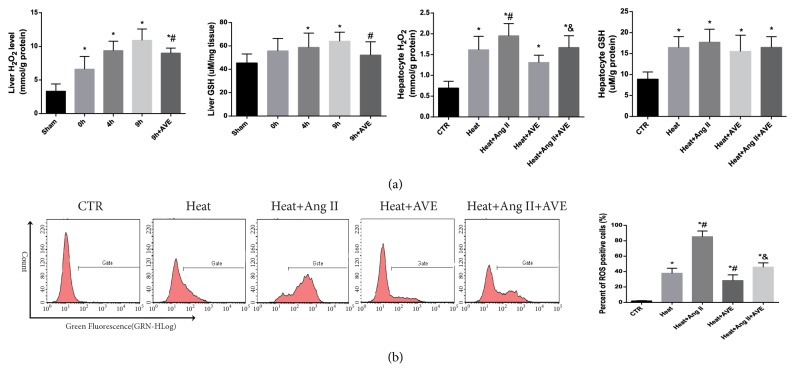
*The effect of AVE 0991 on the production of reactive oxygen species induced by heat stress and Ang II*. (a) The content of hydrogen peroxide and reduced glutathione in the liver tissue and cultured BRL-3A cells. Ang II and/or AVE 0991 were added to the supernatant for an additional 9 h after heat stress. (b) Reactive oxygen species were analysed by flow cytometry. The quantified percentages are shown in the graph (right). *∗P*<0.05 compared with the sham or control groups. #*P*<0.05 compared with the heat group. &*P*<0.05 compared with the heat+Ang II group. The data are presented as the mean ± SD. All of the assays were performed in triplicate.

**Figure 5:**
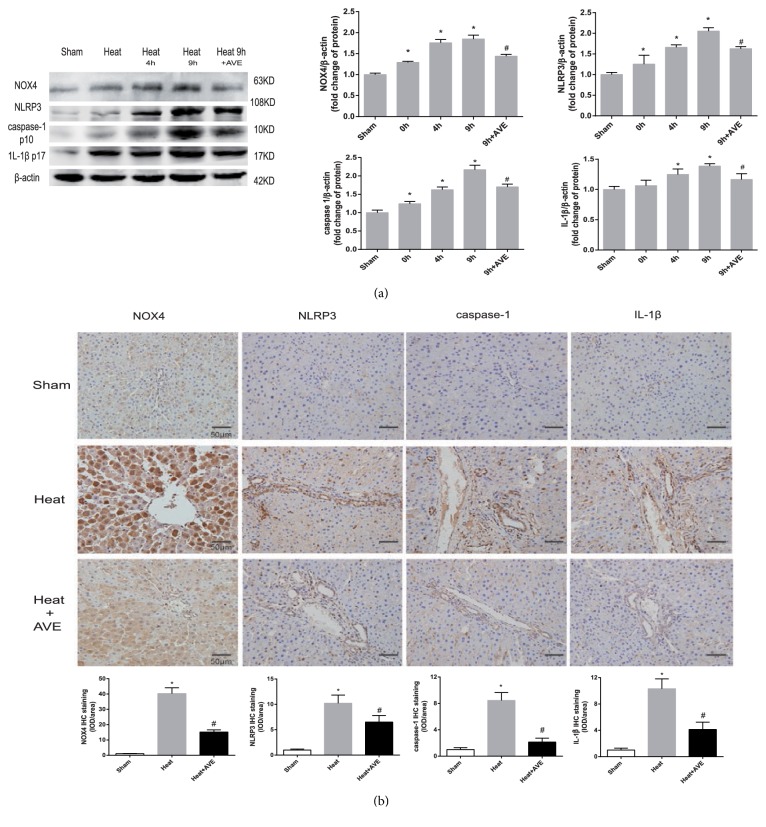
*AVE 0991 inhibited the expression of NLRP3, caspase-1 and IL-1β by downregulating NOX4 in vivo*. (a) Protein expression levels in the liver tissue were analysed by western blotting. The quantified relative protein expression is shown in the graph (right). *∗P* < 0.05 compared with the sham group. #*P*< 0.05 compared with the heat 9 h group. (b) Immunohistochemical staining for NOX4, NLRP3, caspase-1 and IL-1*β* protein expression levels. The comparative fold change of density/area is shown in the graph (bottom). Heat group: 9 h after heatstroke onset. The data are presented as the mean ± SD. All of the assays were performed in triplicate.*∗P* < 0.05 compared with the sham group. #*P*< 0.05 compared with the heat group.

**Figure 6:**
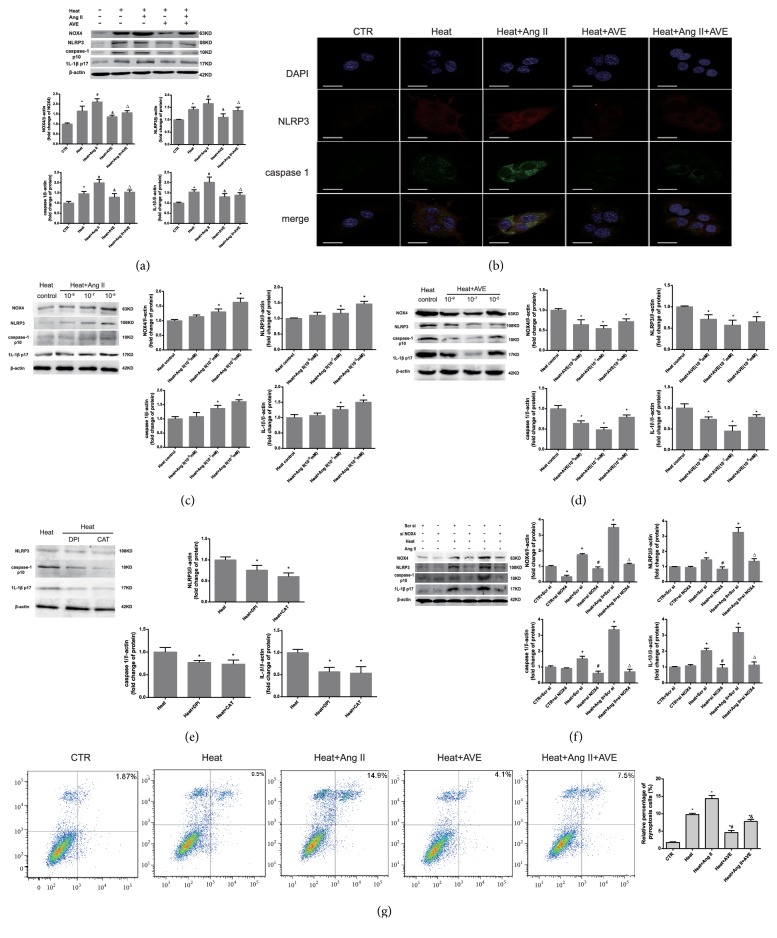
*AVE 0991 attenuated pyroptosis and hepatocyte damage induced by heat stress by inhibiting the ROS-NLRP3 inflammatory signalling pathway in vitro*. (a) The protein expression levels of NOX4, NLRP3, caspase-1 and IL-1*β* in BRL -3A cells were analysed by western blotting. *∗P*<0.05 compared with the control group. #*P*<0.05 compared with the heat group. &*P*<0.05 compared with the heat+Ang II group. (b) Immunofluorescence staining was conducted and observed using confocal laser microscopy. The scale bar indicates 20 *μ*m. NLRP3: red; caspase-1: green. The nuclei stained with DAPI (blue). (c, d) BRL-3A cells subject to heat stress were treated with varying concentrations of Ang II and AVE 0991. The quantified relative protein expression analysed by western blotting is shown in the graph (right). *∗P*<0.05 compared with the heat group. (e) The effect of 10^-5 ^M DPI and 10 mM CAT on BRL-3A cells pre-treated with heat stress. *∗P* < 0.05 compared with the heat group as a control. (f) Protein expression after siRNA transfection targeting NOX4. *∗P*<0.05 compared with the control group. #*P*<0.05 compared with the heat group. △*P*<0.05 compared with the heat+Ang II group. (g) The expression of activated caspase-1 was analysed by flow cytometry. *∗P*<0.05 compared with the sham or control groups. #*P*<0.05 compared with the heat group. &*P*<0.05 compared with the heat+Ang II group. n=6 per group. The data are presented as the mean ± SD. All of the assays were performed in triplicate. DPI: diphenylene iodonium; CAT: catalase.

The H_2_O_2_ content and flow cytometry analysis showed that heat stress induced the extensive production of ROS in the liver tissue and hepatocytes, accompanied by increased levels of the antioxidant GSH (Figures 4(a) and 4(b)). Western blot and IHC analyses showed that the upregulation of NOX4 protein may contribute to this process (Figures 5 and 6(a)). Ang II further upregulated NOX4 expression and increased ROS, which was inhibited by AVE 0991 (Figures 4(b), 5(a), and 6(a)).

### 3.7. AVE 0991 Suppressed the Expression of NLRP3, Caspase-1 and IL-1*β* in Hepatocytes under Heat Stress

*In vivo*, western blot analysis and IHC showed increased protein expression levels of NOX4, NLRP3, caspase-1 and IL-1*β* in the liver tissue of heatstroke rats (Figure 5).* In vitro*, Ang II synergized with the pathogenic effect of heat in a dose-dependent manner, which was antagonized by AVE 0991 (Figures 6(a)–6(d)). Immunofluorescence cytochemistry showed an association between NLRP3 and caspase-1 (Figure 6(b)). ROS scavengers and NOX4 siRNA also suppressed the increases in NLRP3, caspase-1 and IL-1*β* expression levels, demonstrating the key role of NOX4-mediated ROS in this pathway (Figures 6(e) and 6(f)). Activated caspase-1 analysis by flow cytometry showed that pyroptosis was enhanced by Ang II and attenuated by AVE 0991 (Figure 6(g)).

## 4. Discussion

In this study, we found for the first time that the imbalance of Ang II and Ang-(1-7) in heatstroke contributes to liver damage initiated by ROS. Restoring the imbalance using AVE 0991 attenuated liver damage by inhibiting the ROS-NLRP3 signalling pathway. The main findings are summarized as follows. First, enhanced Ang II and attenuated Ang-(1-7) induced by heat stress associated with liver damage were observed. Second, the reversed ratio of Ang II and Ang-(1-7) in patients served as a potential predictive biomarker of hepatic dysfunction. Third, AVE 0991 attenuated liver damage, which was worsened by overproduction of Ang II via the ROS-NLRP3 inflammatory signalling pathway.

Theoretically, RAS is likely to be stimulated by heatstroke, which is a disease commonly associated with fluid loss and a decline in renal perfusion (Noakes et al., 1998). However, the status of RAS in heatstroke is poorly understood, which was the first aim of this study. This study found increased Ang II and decreased Ang-(1-7) in the serum of heatstroke patients, as well as in the serum and liver tissue of heatstroke rats. This result is similar to some other models subjected to acute stress [17, 18]. The change in angiotensin-converting enzymes may be responsible for the conversion. These results provoked our interest in assessing angiotensin as a predictive biomarker in heatstroke.

Emerging evidence supports the value of Ang II and Ang-(1-7) as potential biomarkers, mainly in inflammatory diseases [19, 20]. In addition, considering that liver damage is always delayed by a few days, there are few reliable markers to predict the occurrence and prognosis of liver damage as a result of heatstroke [21, 22]. In this study, we first found that the reversed ratio of Ang II and Ang-(1-7) was associated with hepatic damage and illness severity in heatstroke patients. ROC and Hosmer-Lemeshow tests further demonstrated that both Ang II and Ang-(1-7) were valuable for predicting hepatic damage. The combination of harmful and beneficial angiotensin increased the value of the diagnosis, which is consistent with other studies using a ratio of two relevant indicators [23]. It is speculated that direct stress induced by heatstroke was responsible for the initial change in the Ang II/Ang-(1-7) ratio. Therefore, whether angiotensin is qualified for long-term prognosis and re-evaluation is questionable.

We speculated that the upregulation of Ang II as a result of heatstroke was the compensatory consequence of the maintenance of blood pressure and electrolyte homeostasis. In contrast, excessive production of Ang II could have negative effects in a precarious local microenvironment by mediating inflammation and cellular damage [24, 25]. To the best of our knowledge, the excellent protective effect of Ang-(1-7) as a natural antagonist of Ang II has received increasing attention [26]. However, the short half-life, which is only 9 seconds in rodents, and limited route of administration hinder the advantages of Ang-(1-7). AVE 0991, which is a synthetic analogue of Ang-(1-7), does not possess these disadvantages and has consistent biological effects [27]. In addition, the use of angiotensin II receptor blockers has a safety risk in the case of hemodynamic instability. With these in mind, we used AVE 0991 as the intervention agent for further experiments.

Angiotensin plays a role through the ROS-triggered pathway [28]. In this study, liver inflammation was accompanied by parallel ROS generation, which was reduced by antioxidants after heatstroke. Ang II, which was determined to be the independent and unexpected source of ROS besides heat stress, decreased inflammation. In addition, AVE 0991 antagonizes Ang II inside RAS and exerts a subtle antioxidant effect, which is consistent with Ang-(1-7) [29, 30]. Therefore, we inferred that AVE 0991 ameliorated liver damage in heatstroke mainly through ROS. However, our previous work also found that Ang II activates downstream pathways not only through ROS but also through Spry1/ERK/NF-*κ*B pathways [31], suggesting that the role of other signalling pathways in heatstroke liver requires further investigation.

The upstream signalling mechanisms by which Ang II induces ROS production are complex and unclear, but NAD(P)H oxidases have been considered to be the main source [32]. Consistent with a previous study, our research showed that the upregulation of NOX4 stimulated by Ang II in the liver contributed to increased ROS, which was specifically inhibited by siRNA [33]. However, mitochondrial dysfunction has also been attributed to enhanced ROS in some heatstroke studies [8, 34]. Therefore, the roles of mitochondria and other NAD(P)H oxidases cannot be excluded.

Furthermore, the NLRP3 inflammasome, which is responsible for liver injury after heatstroke, depends on ROS as a major stimulus [6]. Another study also addressed the effect of the ROS-NLRP3 signalling pathway on the activation of caspase-1 and liver inflammation (Lu et al., 2015; [35]). Many efforts have been made to downregulate this pathway by investigating the negative regulator of the NLRP3 inflammasome, either upstream or downstream of ROS. However, few agents provide novel strategies for clinical practice [36]. A previous study showed that exogenous Ang-(1-7) could be a potential negative regulator of the NLRP3 inflammasome in the liver [33]. Our study demonstrated that AVE 0991 played a similar role in livers subjected to heatstroke.

The role of pyroptosis in decreasing hepatocyte damage in livers subjected to heatstroke has been addressed in detail. Pyroptosis may not be the only form of programmed cell death after heat stress, but it is indeed a cause of increased inflammatory reactions [6, 37]. The current results repeatedly verified the presence of pyroptosis in our study, which was associated with Ang II and the NLRP3 inflammasome. In fact, this is not the first time that pyroptosis induced by Ang II has been confirmed, even if no type of inflammasome involvement has been mentioned (Bai et al., 2018). Pharmacological inhibitors of NLRP3, caspase-1 and IL-1*β* are effective in terms of alleviating hepatocyte pyroptosis [6]. In our study, we demonstrated for the first time that the endogenous inhibitor in RAS might prompt a prospective therapeutic strategy.

There are some limitations to the present study. First, this study did not provide a new mechanism underlying liver injury induced by heat stress. This study was only an initial investigation of the role of RAS in this process, as well as proposing a promising strategy for monitoring and therapy. Second, this was not strictly a pharmacological study for assessing the PK/PD effect of AVE 0991 in rodents or humans. Further research is still needed before its clinical application.

**Figure 7:**
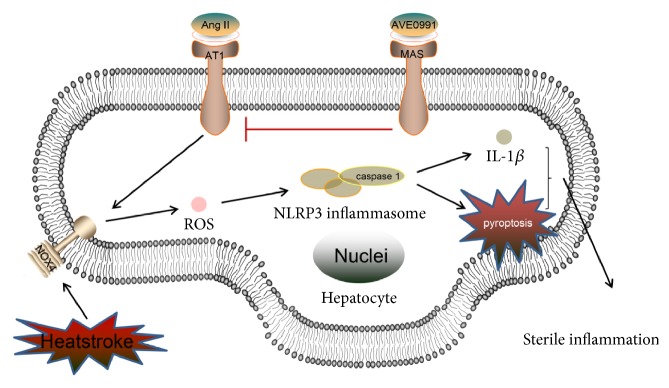
*A schematic of the signalling pathways involved in AVE 0991-attenuated pyroptosis and hepatocyte damage induced by heat stress by inhibiting the ROS-NLRP3 inflammatory signalling pathway*. Ang II: angiotensin II; AT1R: angiotensin II type 1 receptor; ROS: reactive oxygen species; NOX: NADPH oxidase; NLRP3: NOD-like receptor family pyrin domain containing 3; IL-1*β*: interleukin-1*β*.

In summary, the present study demonstrated that the activation of caspase-1 triggered by Ang II-induced ROS contributes to liver damage after heatstroke. AVE 0991 attenuates liver damage by inhibiting the ROS-NLRP3 inflammatory signalling pathway (Figure 7). Consequently, this study revealed the change in Ang-(1-7) and the role of Ang-(1-7) in livers subjected to heatstroke, indicating that AVE 0991 is a potential target for treating liver damage after heatstroke.

## Figures and Tables

**Table 1:** Characteristics of heatstroke patients according to liver function.

Parameters	Hepatic dysfunction (n= 28, 40.6%)	Non-hepatic dysfunction (n=41,59.4%)	*P* value
Ang II (pg/ml)	145.59(44.56-406.98)	39.75(33.32-54.75)	<0.001
Ang-(1-7) (pg/ml)	398.53±257.26	718.59±302.87	<0.001
Ang II/ Ang-(1-7) ratio	0.38(0.12-1.57)	0.07(0.04-0.12)	<0.001
Length of ICU stay (days)	8(2-49)	3(3-5)	<0.001
Organ support (yes/no)	14/14	4/37	-
Death (yes/no)	8/20	0/41	-

Ang II, angiotensin II; Ang-(1-7), angiotensin-(1-7); Organ support: invasive mechanical ventilation, haemopurification or haemodynamic support. Non-normally distributed data are expressed as median (25th percentiles, 75th percentiles) or percentages; “-”, chi-square tests are invalid because of small incidence in the non-hepatic dysfunction group.

**Table 2:** ROC analysis of Ang II, Ang-(1-7), and Ang II/Ang-(1-7) in heatstroke patients used to predict the occurrence of hepatic dysfunction.

Parameters	AUC	*P* value	95% confidence interval	
			Lower Bound	Upper Bound
Ang II	0.780	<0.001	0.669	0.891
Ang-(1-7)	0.804	<0.001	0.599	0.909
AngII/Ang-(1-7)	0.853	<0.001	0.760	0.946

AUC, area under the curve. Ang II, angiotensin II; Ang-(1-7), angiotensin-(1-7).

**Table 3:** Hosmer-Lemeshow tests of Ang II, Ang-(1-7), and Ang II/Ang-(1-7) in heatstroke patients.

Parameters	*χ*2	degree of freedom	*P* value
Ang II	5.079	8	0.749
Ang-(1-7)	8.299	8	0.405
Ang II/ Ang-(1-7)	4.525	8	0.807

Ang II, angiotensin II; Ang-(1-7), angiotensin-(1-7).

**Table 4:** Typical physiological responses in rats during the heat stress.

	Sham group	Heatstroke group
Weight loss (g)	0	14.9±1.2^*∗*^
Maximum core temperature(°C)	36.6±0.2	43.2±0.3^*∗*^
Maximum SBP (mmHg)	112.5±3.8	165.7±8.9^*∗*^

Values are represented as mean±SD.  ^*∗*^*P*<0.05 compared with the sham group.

## Data Availability

The data that support the findings of this study are available from the corresponding author upon reasonable request. The information of patients was not publicly available due to ethical restrictions.
